# Non-Aorto-Ostial Takayasu Arteritis Presenting as Exertional Chest Pain and Syncope Resulting in Coronary Artery Bypass Surgery in a Teenager

**DOI:** 10.7759/cureus.86160

**Published:** 2025-06-16

**Authors:** Megan Gunsaulus, John Kovalchin, Curt Daniels, Hannah Jacobs, Shoghik Akoghlanian

**Affiliations:** 1 Pediatric Cardiology, The Ohio State University, Nationwide Children's Hospital, Columbus, USA; 2 Adult Congenital Heart Disease, The Ohio State University, Nationwide Children's Hospital, Columbus, USA; 3 Cardiovascular Medicine, The Ohio State University Wexner Medical Center, Columbus, USA; 4 Pediatric Rheumatology, The Ohio State University, Nationwide Children's Hospital, Columbus, USA

**Keywords:** cardiac ischemia, coronary artery bypass graft (cabg) surgery, coronary vasculitis, exertional chest pain, pediatric clinical cardiology, pediatric rheumatology, stress cardiac mri, syncope, takayasu arteritis (tak)

## Abstract

Chest pain and syncope are common in adolescents and are usually benign, but they can also be indicative of potentially life-threatening cardiac conditions. We present a rare case of non-aorto-ostial Takayasu arteritis in an adolescent presenting with exertional chest pain and syncope that highlights the importance of considering uncommon diagnoses and conducting comprehensive diagnostic evaluations. The initial diagnostic workup including an ECG and echocardiogram were normal. An ECG stress test demonstrated ST changes consistent with ischemia. Advanced imaging techniques, including cardiac CT and cardiac stress MRI were essential for the accurate diagnosis and management of this patient. Cardiac CT revealed significant wall thickening of the left anterior descending (LAD) artery, consistent with coronary vasculitis, and cardiac stress MRI demonstrated reversible subendocardial ischemia and regional wall motion abnormalities in the LAD distribution. The rheumatologic work-up revealed no additional signs of vasculitis with normal inflammatory markers and unremarkable whole-body MRI. Medical induction therapy with cyclophosphamide and systemic corticosteroids resulted in significant improvement in LAD wall inflammation and thickness. However, the intraluminal LAD diameter remained severely narrowed. Therefore, the patient ultimately underwent coronary artery bypass surgery with a successful outcome and complete resolution of symptoms.

## Introduction

Takayasu arteritis (TA) is an idiopathic, non-specific, and chronic vasculitis that primarily affects young women and is typically characterized by stenosis or dilation of the aorta and its major branches [1]. Isolated coronary TA is rare but has been described in the literature; coronary artery involvement has been reported in 9%-11% of cases by angiographic and histopathologic studies [2,3]. Within these cases, the coronary ostia are the most commonly affected part of the coronary arteries, with involvement seen in 73%-88% of coronary disease patients [3,4]. TA carries significant morbidity and mortality in young patients, particularly when there is coronary involvement, as this increases the risk of myocardial infarction and sudden cardiac death [5].

## Case presentation

**Figure 1:**
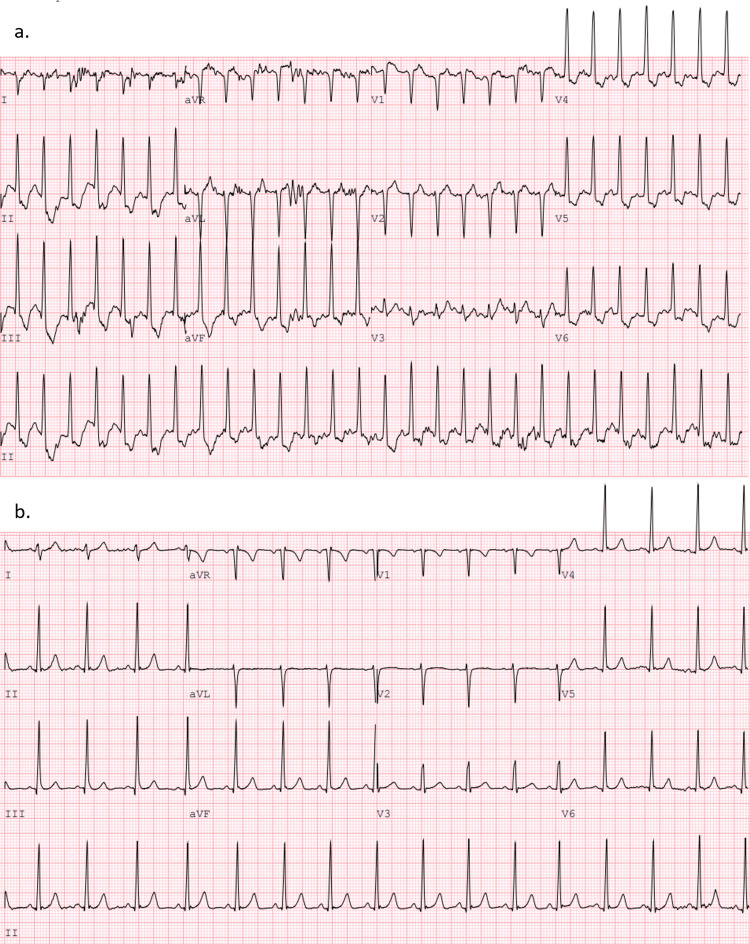
Cardiac stress test at peak exercise demonstrating 3-4 mm inferolateral ST depression (a), which resolved 10 minutes post-exercise (b).

A 16-year-old Caucasian girl presented to the outpatient cardiology clinic for evaluation of exertional chest pain and three episodes of syncope during exercise that were preceded by dizziness. An EKG and Holter monitor test were performed at this visit and the results were normal. An echocardiogram demonstrated no structural heart abnormalities, normal coronary artery origins and proximal courses, and normal biventricular size and systolic function. A Standard Bruce Cardiac Stress Test was subsequently performed. At peak exercise, she appeared pale and diaphoretic and developed severe chest pain, blurred vision, hearing loss, and dizziness with concurrent EKG findings of 3-4 mm inferolateral ST depression. There was no evidence of arrhythmias. Her chest pain and EKG changes persisted for 10 minutes post-exercise (Figure 1). Blood pressure could not be auscultated until mid-recovery.

**Figure 2:**
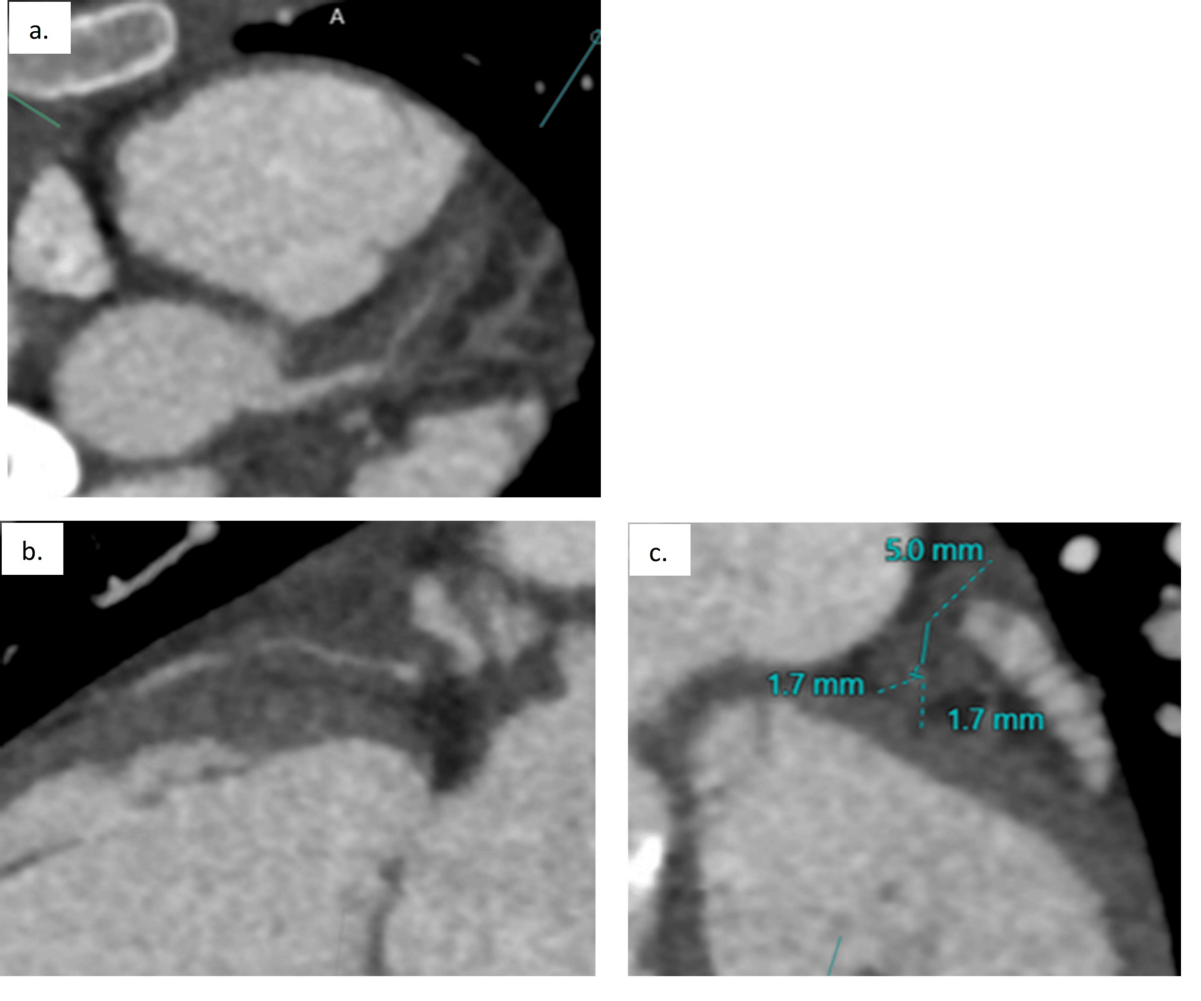
Significant thickening of the left anterior descending (LAD) artery wall (of ~2.5 cm) with severe narrowing of its lumen (a, b). Wall thickening of the distal two-thirds of the left main coronary artery (LMCA) without significant narrowing (a). The LAD diameter was <2 mm, and the surrounding wall thickness measured up to 5 mm (c).

This abnormal stress test prompted counseling for exercise restriction and cardiac CT for further coronary artery evaluation. CT demonstrated normal anatomic origins and courses of the coronary arteries with significant thickening of the left anterior descending (LAD) artery wall resulting in severe narrowing of its lumen. Additionally, there was wall thickening of the distal two-thirds of the left main coronary artery (LMCA) without significant narrowing in this region (Figure 2). There were collateral vessels arising from the circumflex and right coronary artery supplying the distal LAD territory. Of note, the coronary system was left dominant with the circumflex artery terminating in a prominent posterior descending artery (PDA).

The coronary wall thickening was consistent with an inflammatory process, raising concern for vasculitis. The patient was urgently referred to the rheumatology clinic, where she denied any symptoms consistent with vasculitis including fevers, rash, eye symptoms, and pulmonary or renal issues. She had no history to suspect missed Kawasaki disease in her childhood and had never tested positive for COVID-19. Additionally, she had no significant family history of autoimmune disease, vasculopathy, or cardiac disease. The patient underwent an MRI of her head, neck, chest, abdomen, and pelvis, which did not reveal any additional vessel abnormalities (Videos 1, 2).

**Video 1:** Brain MRI of the patient (normal findings).

**Video 2:** Neck, chest, abdomen, and pelvis MRI of the patient (normal findings).

Lab results (Table 1), including a comprehensive metabolic panel, complete blood count, Von Willebrand antigen, complements, and quantitative immunoglobulins, were all normal. She also had negative lupus autoantibodies and anti-neutrophil cytoplasmic antibodies (ANCAs). The antinuclear antibody (ANA) test result was positive. Most notably, her inflammatory markers, including C-reactive protein (CRP) and erythrocyte sedimentation rate (ESR), were within normal limits and remained normal throughout her course.

**Table 1:** Laboratory results from the initial rheumatology visit BUN: blood urea nitrogen; ALT: alanine transaminase; AST: aspartate aminotransferase; WBC: white blood cell; RBC: red blood cell; MCV: mean corpuscular volume; MCH: mean corpuscular hemoglobin; MCHC: mean corpuscular hemoglobin concentration; RDW: red cell distribution width; anti-SM: anti-Smith antibodies; anti-RNP: anti-ribonucleoprotein antibodies; anti-SSA: anti-Sjogren's syndrome A; anti-SSB: anti-Sjogren's syndrome B; ANCA IFA: anti-neutrophil cytoplasmic antibody testing by indirect immunofluorescence assay; AB: antibody; ANA: antinuclear antibody

Laboratory test	Patient's result	Normal range
Sodium	138	135-145 mmol/L
Potassium	4.1	3.6-4.9 mmol/L
Chloride	103	98-110 mmol/L
Carbon dioxide	23	21-30 mmol/L
BUN	8	5-18 mg/dL
Creatinine	0.57	0.5-0.8 mg/dL
Glucose	89	60-115 mg/dL
Calcium	9.3	8-10.5 mg/dL
Total protein	8.2	6.5-8.6 g/dL
Albumin	4.8	3.4-5.2 g/dL
ALT	15	<36 U/L
AST	19	15-50 U/L
Alkaline phosphatase	75	59-126 U/L
Bilirubin total	0.5	0.1-1.0 mg/dL
WBC	8.5	4.5-13.0 × 10^3^/uL
RBC	4.7	4.1-5.1 × 10^6^/uL
Hemoglobin	12.9	12.0-16.0 g/dL
Hematocrit	38.4	36.0%-46.0%
MCV	82.2	78.0-102.0 fL
MCH	27.6	25.0-35.0 pg
MCHC	33.6	31.0%-37.0%
RDW	13.5	10%-14.2%
Platelet count	394	142-508 × 10^3^/uL
Von Willebrand antigen	88	50%-150%
C3 complement	96	90-154 mg/dL
C4 complement	18	15-42 mg/dL
IgG	1082	487-1327 mg/dL
IgA	145	60-337 mg/dL
IgM	152	49-201 mg/dL
IgE	180	0-257 IU/mL
Anti-SM	<20	<20 units
Anti-RNP	<20	<20 units
Anti-SSA	<20	<20 units
Anti-SSB	<20	<20 units
ANCA IFA pattern	None detected	N/A
ANCA IFA titer	<1:20	<1:20
Serine proteinase 3, IgG	1	0-19 AU/mL
Myeloperoxidase AB, IgG	0	0-19 AU/mL
ANA screen	Positive	N/A
ANA titer	1:320	N/A
Sedimentation rate	10	<20 mm/h
C-reactive protein	<0.5	<1.0 mg/dL

**Figure 3:**
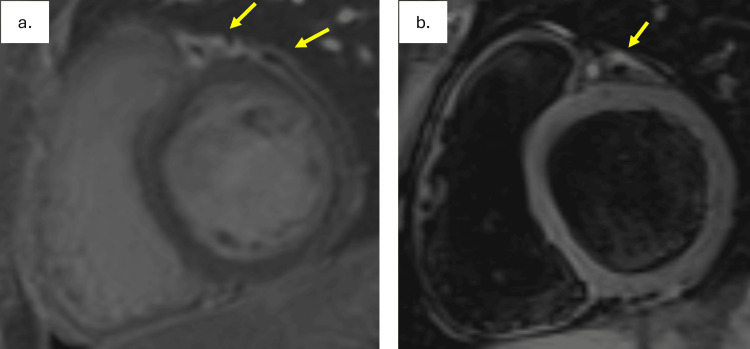
(a) No myocardial late gadolinium enhancement (LGE) was detected. There was LGE of the epicardial fat surrounding the proximal left anterior descending (LAD) artery with contiguous extension into the adjacent parietal pericardium (arrows), likely reflecting local inflammatory changes related to peri-coronary vasculitis. (b) T2-weighted imaging demonstrated evidence of inflammation and edema surrounding the proximal LAD (arrow).

The patient was subsequently diagnosed with isolated coronary vasculitis (non-aorto-ostial Takayasu arteritis) and admitted for induction therapy with cyclophosphamide and methylprednisolone followed by prednisone weaning, as an outpatient. During this admission, cardiac MRI was performed and demonstrated no evidence of myocardial late gadolinium enhancement. There was mild enhancement of the epicardial fat surrounding the LAD with extension into the visceral pericardium, likely reflecting local inflammatory changes. T2-weighted imaging demonstrated hyperintense signal consistent with inflammation and edema surrounding the proximal LAD (Figure 3).

**Figure 4:**
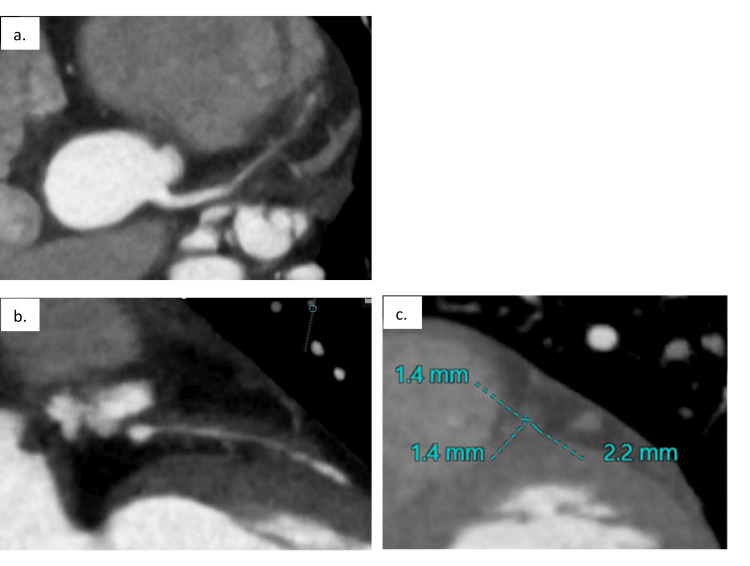
Cardiac CT completed after receiving a cumulative dose of 6,400 mg cyclophosphamide. Compared to the prior CT, the left anterior descending (LAD) artery wall thickness was significantly improved, but the intraluminal diameter was unchanged (a, b). The LAD diameter remained <2 mm, and the surrounding wall thickness measured 2.2 mm (c).

After this admission, the patient underwent monthly cyclophosphamide infusions. Four months following induction therapy, after completing four additional cyclophosphamide infusions, a repeat cardiac CT scan was performed. Compared to the prior CT, the LAD wall thickness was significantly improved, but the intraluminal diameter of the LAD was unchanged and remained severely narrowed (Figure 4).

The patient completed a cumulative dose of 9,400 mg cyclophosphamide over a period of seven months. She was then started on a tumor necrosis factor inhibitor agent (Hyrimoz) and low-dose methotrexate for maintenance therapy in addition to her previously established prednisone wean. At this time, cardiac stress MRI using regadenoson was conducted to assess risk, and guide future management and exercise restriction recommendations. This study demonstrated significant, reversible subendocardial ischemia of the inferoseptal, anteroseptal, and anterior walls of the base and mid-left ventricle and the inferoseptal segment of the apex (Video 3). These regions corresponded with the territory perfused by the LAD.

**Video 3:** After the administration of regadenoson (a, top row), there was notable subendocardial ischemia of the inferoseptal, anteroseptal, and anterior walls of the base and mid-left ventricle and the inferoseptal segment of the apex (arrows). After regadenoson reversal with aminophylline (b, bottom row), this was no longer evident, suggestive of reversible ischemia.

Additionally, there was associated significant regional wall motion abnormalities involving the interventricular septum (Video 4). The perfusion defects and regional wall motion abnormalities resolved following regadenoson reversal with aminophylline.

**Video 4:** After the administration of regadenoson (a, top row), there was significant regional wall motion abnormality involving the interventricular septum. After regadenoson reversal with aminophylline (b, bottom row), the left ventricular function appeared normal with no wall motion abnormality.

The patient’s case was ultimately discussed during a multidisciplinary case management conference. The consensus at this time was to refer the patient to adult interventional cardiology for cardiac catheterization with coronary angiography and possible transcatheter intervention. On angiography, the patient was found to have severe diffuse narrowing throughout the LAD with 95% stenosis at the LAD origin and 100% stenosis of the mid-LAD. The distal LAD was supplied by collaterals primarily from the right coronary artery. The distal left circumflex was 85% stenosed (Video 5). She also had an elevated left ventricular end diastolic pressure (LVEDp) of 16 mmHg.

**Video 5:** Severe diffuse narrowing throughout the left anterior descending (LAD) artery with 95% stenosis at the LAD origin and 100% stenosis of the mid-LAD (top two arrows). The distal LAD was supplied by collaterals primarily from the right coronary artery. The distal left circumflex was 85% stenosed (bottom left arrow).

Following completion of her steroid wean, the patient underwent coronary artery bypass surgery (CABG) of the left anterior descending, left posterior descending, and ramus intermedius coronary arteries with the use of the right and left internal mammary arteries and saphenous vein. Her post-operative course was complicated by a pericardial effusion with tamponade physiology requiring a pericardial window approximately one month after surgery. She has been regularly exercising without any chest pain or anginal equivalent. A stress echo was performed approximately five months post-operatively and demonstrated subtle septal hypokinesis with exercise but was otherwise unremarkable. This finding did not appear to align with any major coronary territory and is commonly observed following CABG [6]. She continues on Hyrimoz and methotrexate.

## Discussion

This case highlights a rare and significant presentation of non-aorto-ostial Takayasu arteritis in an adolescent female presenting with exertional chest pain and syncope. Pediatric chest pain and syncope present a paradox, as they are common and benign in most cases, yet can also be key symptoms of rare and life-threatening cardiac conditions [7]. It is therefore critical to differentiate benign conditions from more serious cases, especially when symptoms occur with exertion, which raises concern for underlying pathology. Chest pain and syncope may be the first manifestations of the underlying cardiovascular pathology such as cardiomyopathy, congenital coronary anomalies, or in rare cases, inflammatory diseases like coronary vasculitis [8]. Careful evaluation and timely diagnostic workup are essential to identify any potential underlying cardiac causes, particularly when symptoms are triggered by physical activity.

This case emphasizes the importance of considering a cardiac stress test when evaluating exertional chest pain and syncope. If symptoms persist despite an unremarkable EKG and echocardiogram, further work-up with an ECG stress test should be considered. Had this further testing not been pursued with our patient, the diagnosis may have been missed, leading to false reassurance despite an increased risk of sudden cardiac death. Our patient’s stress test revealed chest pain and pre-syncopal symptoms with ST segment changes. The pre-syncopal symptoms during this test, along with her history of syncope with exercise, could potentially be attributed to a vagal response to chest pain, hypotension, or ischemic ventricular arrhythmia, although the latter was not observed during the stress test itself. Advanced imaging techniques, including cardiac CT and stress MRI, were also essential diagnostic tools in this case. The initial cardiac CT was pivotal in diagnosing coronary vasculitis in this patient, particularly in the context of normal inflammatory markers. The cardiac stress MRI demonstrated reversible ischemia in the region of the LAD, confirming that the patient’s symptoms of chest pain were likely consistent with ischemia. This finding played a crucial role in risk stratification and influenced the decision to pursue surgical intervention.

Data on CABG outcomes in TA is limited due to the rarity of the disease, with current guidelines recommending invasive therapy only for life- or organ-threatening cases or when a patient’s activities are significantly impacted. Surgery should also be avoided during active disease [9]. A recent study compared revascularization to medical therapy in patients with TA and coronary artery involvement and found that revascularization improved heart function and reduced cardiovascular events more effectively than medical therapy alone. This is particularly pertinent when significant coronary stenosis or occlusion is present [10]. Guided by these findings, surgical intervention was recommended for this patient. She subsequently underwent successful revascularization with resolution of symptoms of exertional chest pain postoperatively.

The long-term outcomes of revascularization in TA patients remains uncertain. Fan et al. conducted a comprehensive analysis of childhood-onset TA, noting that while early intervention improves prognosis, the disease can persist or relapse over time. These findings emphasize the need for long-term follow-up as vascular changes can continue even after surgical revascularization [11]. Thus, close monitoring for disease progression and further vascular complications is essential for optimal long-term management.

## Conclusions

This case emphasizes the importance of considering coronary vasculitis, specifically non-aorto-ostial Takayasu arteritis, in the differential diagnosis of exertional chest pain and syncope in a young patient. Timely recognition of coronary artery disease in the pediatric population, despite the rarity of the condition, is critical to preventing life-threatening cardiovascular events. Treadmill stress testing, cardiac CT, and cardiac stress MRI were essential for the accurate diagnosis and management of this patient. In the setting of limited and variable data pertaining to the outcomes of CABG in TA patients, this case adds to the body of evidence, demonstrating a successful initial outcome with complete resolution of chest pain following surgical intervention.
